# Fibroma of tendon sheath of the hand in a 3-year-old boy: a case report

**DOI:** 10.1186/s12891-020-03728-x

**Published:** 2020-11-10

**Authors:** Hiroki Shibayama, Yuichiro Matsui, Daisuke Kawamura, Atsushi Urita, Chikako Ishii, Tamotsu Kamishima, Mutsumi Nishida, Ai Shimizu, Norimasa Iwasaki

**Affiliations:** 1 Department of Orthopedic Surgery, Faculty of Medicine and Graduate School of Medicine, Hokkaido University, Kita-15 Nishi-7, Kita-ku, Sapporo, Hokkaido 060-8638 Japan; 2 Faculty of Health Sciences, Hokkaido University, Kita-12 Nishi-5, Kita-ku, Sapporo, Hokkaido 060-0812 Japan; 3 Division of Laboratory and Transfusion Medicine / Diagnostic Center for Sonography, Hokkaido University Hospital, Kita-14, Nishi-5, Kita-ku, Sapporo, Hokkaido 060-8648 Japan; 4 Department of Surgical Pathology, Hokkaido University Hospital, Kita-14 Nishi-5, Kita-ku, Sapporo, Hokkaido 060-8648 Japan

**Keywords:** Fibroma of tendon sheath, Soft tissue tumor of a young child’s hand, Preoperative imaging study, Case report

## Abstract

**Background:**

Fibroma of tendon sheath (FTS) is a rare benign soft tissue tumor that often occurs in the upper extremities. It manifests as a slow-growing mass, often without tenderness or spontaneous pain. FTS occurs most commonly in people aged 20–40 years and is extremely rare in young children. Because FTS presents with atypical physical and imaging findings, it might be misdiagnosed as another soft tissue tumor such as a ganglion cyst or tenosynovial giant cell tumor (TSGCT). Although marginal resection is usually performed, a high rate of local recurrence is reported.

**Case presentation:**

A boy aged 3 years and 1 month visited our outpatient clinic with a complaint of a mass of the left hand. An elastic hard mass approximately 20 mm in diameter could be palpated on the volar side of his left little finger. This mass was initially diagnosed as a ganglion cyst at another hospital. Ultrasonography revealed a well-circumscribed hypoechoic mass with internal heterogeneity on the flexor tendon. On magnetic resonance imaging (MRI), the mass showed iso signal intensity to muscle on T1-weighted images, and homogeneously low signal intensity to muscle on T2-weighted images. The mass was peripherally enhanced after contrast administration. FTS was initially suspected as the diagnosis on the basis of these imaging features. Because of the limited range of motion of his little finger, surgery was performed when he was 4 years old. Histopathological findings indicated the mass was well-circumscribed and contained scattered spindle cells embedded in a prominent collagenous matrix. The spindle cells contained elongated and cytologically bland nuclei with a fine chromatin pattern. Nuclear pleomorphism and multinucleated giant cells were not observed. On the basis of these findings, we made a diagnosis of FTS. One year after surgery, no signs of local recurrence were observed.

**Conclusions:**

We experienced an extremely rare case of FTS in the hand of a 3-year-old child. We especially recommend ultrasonography for hand tumors of young children to diagnose or eliminate ganglion cysts. MRI helped differentially diagnose FTS from TSGCT. Although marginal resection can be performed as a treatment, great care should be taken postoperatively because FTS has a high possibility of local recurrence.

## Background

Fibroma of tendon sheath (FTS) is a rare benign soft tissue tumor that often occurs in the upper extremities. In 1923, Buxton classified benign soft tissue tumors of the tendon sheath into the following four categories: lipoma, fibroma, chondroma, and ganglion [1]. FTS was first described in detail by Geshickter and Copeland in 1949 [2]. This fibroma manifests as a slow-growing mass, often without tenderness or spontaneous pain. It is difficult to distinguish from tenosynovial giant cell tumor (TSGCT) based on clinical symptoms and imaging examination. Satti MB reported that FTS had advanced hyalinization of TSGCT by microscopy and might be a continuous pathological condition [3]. Although FTS tends to strongly adhere to the tendon or tendon sheath, marginal resection is usually performed. Therefore, careful attention should be paid postoperatively because of the high local recurrence rate after surgery, which was 24% in a previous report [4]. FTS occurs most commonly in persons aged 20–40 years and is extremely rare in early childhood. Here we report a case of FTS in the hand of a 3-year-old boy.

## Case presentation

A boy aged 3 years and 1 month visited our outpatient clinic with a complaint of a mass of the left hand. There were no problems with his delivery, medical history, or family history. At the age of 2 years and 7 months, his parents noticed a protuberance on the volar side of his left hand. There was no apparent episode of trauma. The lesion was diagnosed as a ganglion cyst at a nearby hospital and he was followed-up. However, the mass continued to grow, and further examination was recommended at a health screening when he was 3 years old.

**Fig. 1:**
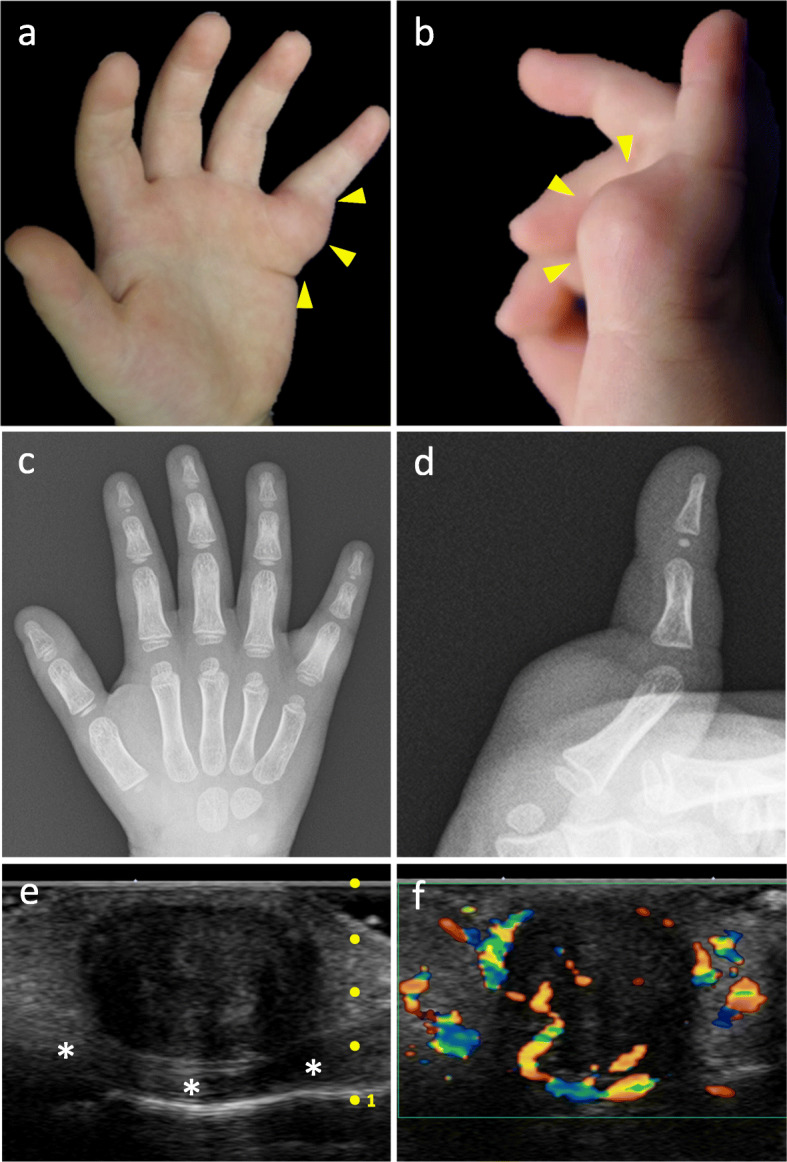
**a**, **b** The mass (yellow arrowheads) is located on the volar aspect of the left little finger from the distal palmer crease to the proximal interphalangeal crease. **c, d** Anteroposterior and lateral radiographs show a circumferential soft tissue shadow on the proximal phalange of the left little finger. Bony abnormalities are not observed. **e** Ultrasonography demonstrates a well-circumscribed heterogeneous low echoic lesion. The lesion is located on the flexor tendon (white asterisks) and the boundary is clearly maintained. **f** Color Doppler ultrasonography shows blood flow into the lesion

**Fig. 2:**
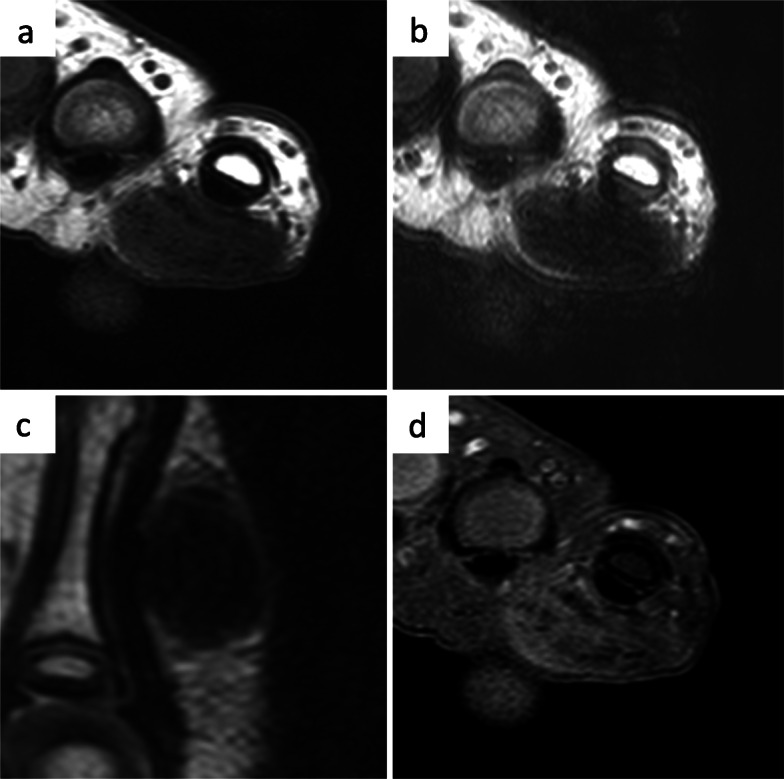
Preoperative magnetic resonance images. The mass shows **a** iso signal intensity to muscle on T1-weighted axial images and **b**, **c** homogenous and low signal intensity to muscle on T2-weighted axial and sagittal images, respectively. **d** The mass is peripherally enhanced in T1-weighted fat saturation axial images with gadolinium enhancement

On physical examination, an elastic hard mass approximately 20 mm in diameter was palpated on the volar side of the left little finger from the distal palmar crease to the proximal interphalangeal crease (Fig. 1a-b). There was no redness, tenderness, or adhesion to the skin, but it had adhered to the basal tissue. The range of metacarpophalangeal joint flexion of the left little finger was 70°, whereas it was 90° for the right little finger. Plain X-ray images showed a soft tissue shadow without calcification or bony alteration (Fig. 1c-d). Ultrasonography revealed a well-circumscribed hypoechoic mass with internal heterogeneity on the flexor tendon (Fig. 1e). Blood flow, signaling flow into the mass, was observed by color Doppler imaging (Fig. 1f). Because a cystic lesion such as a ganglion cyst was unlikely and a solid tumor was considered, magnetic resonance imaging (MRI) was performed under general anesthesia. On MRI, the mass showed iso signal intensity to muscle on T1-weighted images (T1WI) containing low signal intensity bands (Fig. 2a) and homogeneously low signal intensity to muscle on T2-weighted images (T2WI) (Fig. 2b-c). It was peripherally enhanced on gadolinium-enhanced T1WI with fat saturation. Non-enhancing low signal intensity bands were seen inside the mass (Fig. 2d). Differential diagnosis considered FTS and TSGCT on the basis of its location and signal properties, an imaging diagnosis of FTS was made because the lesion showed homogeneous, low signal intensity on T2WI and relatively weak, peripheral contrast enhancement, compatible with the diffuse and rich proliferation of collagen.

**Fig. 3:**
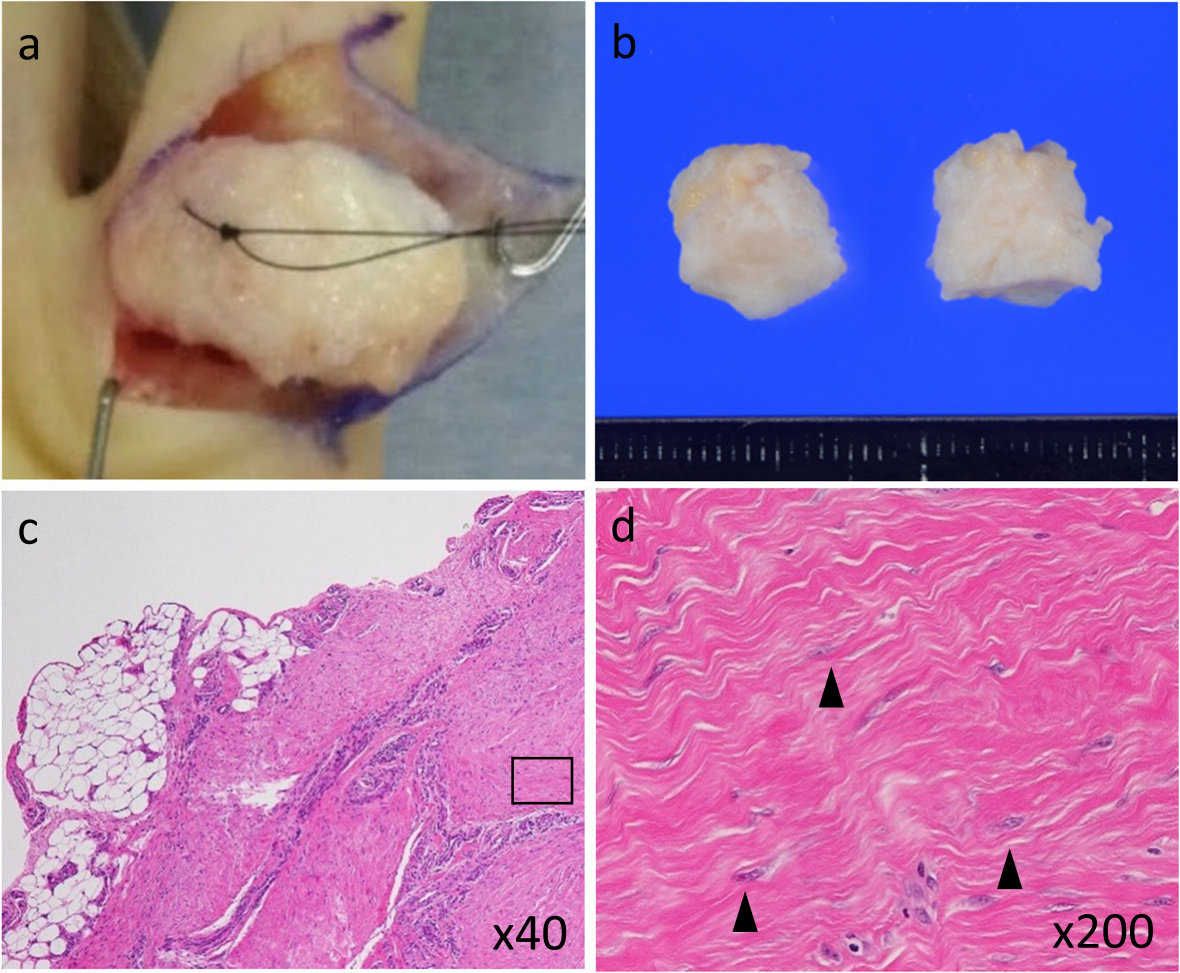
**a** Intraoperative photograph. The mass was dissected easily as there was no strong adhesion to the skin, tendon sheath, or neurovascular bundles. **b-d** Gross and microscopic findings for the resected surgical specimen. **b** The mass is circumferential and yellowish-white (the mass is divided into two pieces). **c** Hematoxylin and eosin (H&E) staining of the mass sections (magnification 40×). The mass is well-circumscribed and contains abundant fibrous tissues. **d** H&E staining of mass section (magnification 200×). The spindle cells contain elongated and cytologically bland nuclei with a fine chromatin pattern (black arrowheads). Nuclear pleomorphism and multinucleated giant cells were not observed

Because of the restricted range of motion and the increased difficulty of using his left hand in daily life, we decided to perform surgical resection after obtaining informed consent from his parents. The surgery was performed under general anesthesia when he was 4 years old. After a zigzag skin incision, a yellowish-white mass was found below the skin. There was no obvious adhesion to the surrounding tissues including the skin, flexor tendon, and neurovascular bundles (Fig. 3a). The mass size was 22 × 11 × 10 mm (Fig. 3b). Skin closure was performed without the need for any special technique.

Microscopically, the excised mass was well-circumscribed and contained scattered spindle cells embedded in a prominent collagenous matrix (Fig. 3c). A high-power view revealed spindle cells with elongated, cytologically bland nuclei in a fine chromatin pattern. Necrosis, nuclear pleomorphism, mitoses, multinucleated giant cells, hemosiderin, and eosinophilic cytoplasmic inclusion bodies were not observed (Fig. 3d). Immunohistochemistry demonstrated the tumor cells were focally positive for α-SMA, and negative for desmin, CD34, and KIT (CD117). There was no nuclear staining for β-catenin. On the basis of these findings, a diagnosis of FTS was made. One year after surgery, no signs of local recurrence were observed. The range of motion of the left little finger was the same as the right little finger and the patient could use his left hand without any difficulties.

## Discussion and conclusions

FTS is a rare benign, slow-growing soft tissue tumor with a high rate of local recurrence following surgical resection [4]. Approximately 80% of FTS occurs in the upper extremities such as the wrist, hand, or fingers [5]. For the fingers, 85% of the cases occur in the thumb, index, and middle finger. The ring and little fingers are infrequently involved for unknown reasons [4]. With regard to the prevalence of FTS, Millon et al. reported that among 208 cases of soft tissue tumors in the hand, FTS occurred in only 7 cases (3.4%) [6]. FTS is more common in males than females, with a ratio of 3:1, and mostly occurs in patients aged from 20 to 40 years [4]. There have been few reports of FTS in young children aged under 9 years [4, 5, 7, 8]. Because of its atypical physical and imaging findings, FTS might be misdiagnosed as a soft tissue tumor such as a ganglion cyst or TSGCT.

There have been five reports of cytogenetic analysis to determine the causes of FTS. Dal Cin et al. investigated whether FTS represented a reactive fibrosing process or a type of neoplasm [9]. They found a chromosomal abnormality characterized by a t(2;11)(q31–32;q12) was present clonally. This finding suggests that FTS has the properties of a neoplasm. However, Carter et al. confirmed USP6 genetic rearrangements, which are often recognized in nodular fasciitis, in 6 of 9 FTS cases [10]. This suggests that FTS might have the same pathology as nodular fasciitis. Although three of six chromosomal abnormalities involved the long arm of chromosome 11 in three other reports, their clinical significance is still unknown [11–13]. Thus, further molecular cytogenic analysis is needed to clarify the etiology of FTS.

Among the four reports of FTS in young children, one presented a case of FTS that occurred in the patella tendon of a 6-year-old boy [8], and the others described a case series with no details related to the site of occurrence, symptoms, and examination findings [4, 5, 7]. Here, we report an extremely rare case of FTS that occurred in the hand of a 3-year-old child. Approximately 10% of FTS develop following trauma [12]. In our case, this tumor occurred without any apparent traumatic history and the cause of the occurrence was unclear. FTS often has strong adhesion to the skin, tendon, tendon sheath, or neurovascular bundles [3]. However, we found no obvious adhesion between the tumor and the surrounding tissues in this early-onset case.

The differential diagnosis includes ganglion cyst, infantile digital fibromatosis, and TSGCT. First, ganglion cysts can be easily diagnosed by ultrasonography. Therefore, we recommend ultrasonography as the primary image analysis when a subcutaneous soft tissue mass is suspected clinically, especially for young children. Second, infantile digital fibromatosis is also a rare soft tissue tumor and the lesion is often painful and covered by shiny red skin [14]. In our case, such physical findings were absent and the histopathology revealed no eosinophilic cytoplasmic inclusion bodies, which are specific to infantile digital fibromatosis. Third, MRI differentially diagnosed FTS from TSGCT. According to literature reviewed by Moretti et al., the appearance of FTS on T2WI is variable: some reports describe a heterogeneous mass with mixed areas of low and high signal intensities and others homogeneously low signal intensity [15]. In our case, FTS was suspected due to homogeneously low signal intensity on T2WI. TSGCT seemed less likely as it typically presents with small, scattered foci of low signal intensity on T1WI and T2WI due to the presence of hemosiderin [16]. In addition, the lack of mild enhancement makes FTS more likely than TSGCT [17]. In our case, there was relatively weak, peripheral contrast enhancement with low intensity bands, favoring a diagnosis of FTS. However, histopathological assessment was essential for an accurate diagnosis.

Regarding the treatment of FTS, marginal resection is typically performed as a surgical procedure. While some authors reported that the indications for the surgical treatment of patients with FTS included symptoms such as pain or a limited range of motion [18], other authors reported that lesions should be removed before any symptoms appear [19]. FTS tends to adhere strongly to the surrounding tissues and is more likely to occur in the hand where functional tissues such as tendons, vessels and nerves are gathered. Therefore, the extent of the resection, with or without adherent tissues, may be a problem for surgery [20]. To prevent the recurrence of FTS, the lesion should be resected with the adherent tissues. Indeed, previous reports of two cases of FTS stated lesions were resected including the digital nerve or radial artery [6, 21]. Therefore, the balance between surgical cure and postoperative dysfunction should be considered for each case [22]. Regarding younger cases, Nason et al. reported that FTS should not be excised with the surrounding functional tissues because it is a benign tumor and the risk of malignant degeneration is extremely low [23]. In 7 cases of FTS under the age of 20 years, only 2 had symptoms other than swelling [6, 8, 13, 23–26]. In the current study, the patient was very young (3 years old) and surgical resection was performed because of the limited range of motion of the finger. Fortunately, there was no adhesion to the surrounding tissues, so we could perform resection of the whole lesion without functional impairment.

Histopathologically, FTS is composed of spindle-shaped cells similar to fibroblasts in a dense collagenous stroma. Cytological atypia is generally not seen. It was reported that the spindle-shaped cells are myofibroblasts and that fibrous tissues have various levels of hyalinization [3]. FTS is characterized by gaps termed “slit-like spaces” that are cavities of blood vessels [4]; however, some cases lack such gaps. Our case presented with collagenous tissues containing scattered spindle cells with fine chromatin and elongated nuclei, but hyalinization and slit-like spaces were not observed. TSGCT contains multinucleated giant cells, foamy histiocytes, mononuclear cells, and hemosiderin [3] but these characteristic findings of TSGCT were not present in our case. Thus, a final diagnosis of FTS was confirmed.

In 1979, Chung and Enzinger reported that the local recurrence rate in 40 patients was 24% at a mean follow-up of 4.3 years after surgical excision [4]. Since then, many studies have reported low or zero local recurrence rates, but these rates may be artifactual because of inadequate follow-up periods [21, 27]. In our case, marginal resection was performed carefully using an air tourniquet and a surgical loupe to reduce the risk of local recurrence. One year after surgery, the patient has no subsequent pain or sign of local recurrence. Further careful follow-up is essential even if there appears to be no local recurrence.

## Data Availability

All data concerning the case are presented in the manuscript.

## Abbreviations

FTS: Fibroma of tendon sheath
TSGCT: Tenosynovial giant cell tumor
MRI: Magnetic resonance imaging
T1WI: T1-weighted images
T2WI: T2-weighted images
